# Supplementary material for: MUG Mel3 Cell Lines Reflect Heterogeneity in Melanoma and Represent a Robust Model for Melanoma in Pregnancy
Source: Int J Mol Sci. 2021 Oct 20;22(21):11318. doi: 10.3390/ijms222111318 (PMC8583216; doi:10.3390/ijms222111318)
Supplement: Supplementary file 1 [file ijms-22-11318-s001.zip › ijms-1380692-supplementary.pdf]

Supporting information

MUG Mel3 cell lines reflect the heterogeneity in melanoma and represent a robust model for melanoma in pregnancy

Silke Schrom[1], Thomas Hebesberger[1], Stefanie Angela Wallner[1], Ines Anders[1], Erika Richtig[2], Waltraud Brandl[3], Birgit Hirschmugl[3,11], Mariangela Garofalo[4], Claudia Bernecker[5], Peter Schlenke[5], Karl Kashofer[6], Christian Wadsack[3,11], Ariane Aigelsreiter[6], Ellen Heitzer[7], Sabrina Riedl[8,11,12], Dagmar Zweytick[8,11,12], Nadine Kretschmer[9], Georg Richtig[10] and Beate Rinner[1, 11*]

[1]Division of Biomedical Research, Medical University of Graz, Graz, Austria

[2]Department of Dermatology, Medical University of Graz, Graz, Austria

[3]Department of Obstetrics and Gynecology, Medical University of Graz, Graz, Austria

[4]Department of Pharmaceutical and Pharmacological Sciences, University of Padova, Padova, Italy

[5]Department of Blood Group Serology and Transfusion Medicine, Medical University of Graz, Graz, Austria

[6]Institute of Pathology, Medical University of Graz, Graz, Austria

[7]Institute of Human Genetics, Diagnostic and Research Center for Molecular BioMedicine, Medical University of Graz, Graz, Austria

[8] Institute of Molecular Biosciences, Biophysics Division, University of Graz, Graz, Austria

[9]Institute of Pharmaceutical Sciences, Department of Pharmacognosy, University of Graz, Graz, Austria

[10]Division of Oncology, Medical University of Graz, Graz, Austria

[11]Bio-TechMed-Graz, Graz, Austria

[12]BioHealth, Graz, Austria

Table S1: **STR profiling.** STR profile for 16 loci are shown for comparison of tumor tissue (pigmented (P) and non-pigmented (NP)) to respective cell lines. Numbers in brackets describe losses for higher passages.

STR-Locus	D3S1358	TH01	D21S11	D18S51	Penta E	D5S818	D13S317	D7S820
Tumor tissue NP	14, 15	7, 8	30, 31	16, 18	7, 14	12, 13	11, 14	8, 12
MUG Mel3 PF, p12, p54	14, 15	7, 8	30, 31	16, 18	7, 14	12, 13	11, 14	8, 12
MUG Mel3 Ph, p5, p48	14, 15	7, 8	30, 31	16, 18	7, 14	12, 13	11, 14	8, (12)
MUG Mel3 NPF, p9, p48	14, 15	7, 8	30, 31	16, 18	7, 14	12, 13	11, 14	8, 12
MUG Mel3 NPh, p7, p47	14, 15	7, 8	30, 31	16, 18	7, 14	12, 13	11, 14	8, 12

STR-Locus	D16S539	CSF1PO	Penta D	Amelogenin	vWA	D8S1179	TPOX	FGA
Tumor tissue P	13	10, 13	9, 13	X	16, 17	13	8, 9	22, 25
Tumor tissue NP	12, 13	10, 13	9, 13	X	16, 17	13	8, 9	22, 25
MUG Mel3 PF, p12, p54	13	10, 13	9, 13	X	16, 17	13	8, 9	22, 25
MUG Mel3 Ph, p5, p48	13	10, 13	9, 13	X	16, 17	13	8, 9	22, 25
MUG Mel3 NPF, p9, p48	12, 13	10, 13	9, 13	X	16, 17	13	8, 9	22, 25
MUG Mel3 NPh, p7, p47	(12), 13	10, 13	9, 13	X	16, 17	13	8, 9	22, 25

Table S2: Selected ICC markers for melanoma cell line characterization. ICC markers HMB45, Melan-A, Tyrosinase, and melanoma chondroitin sulfate proteoglycan (MCSP) are listed with order number and dilutions used for staining of MUG Mel3 cell lines.

Antibody	Clone	Company	Order Number	Dilution	Isotype	Staining
Melanosome	HMB45	Agilent Dako	M0634	1:500	mouse IgG1	cytoplasmic
Melan-A	A103	Agilent Dako	M7196	1:200	mouse IgG1	cytoplasmic / perinuclear
Tyrosinase	T311	Agilent Dako	M3623	1:100	mouse IgG2a	cytoplasmic and / or perinuclear
NG2 / MCSP	LHM-2	R&D Systems	MAB2585	1:200	mouse IgG1	plasma membrane

Table S3: Forward and revers primers used for qPCRs.

Gene	Forward (5´ - 3´)	Reverse (5´ - 3´)	Product length
Melan-A	ACAGTGATCCTGGGAGTCTTAC	TTGAAGAGACACTTTGCTGTCC	168 bp
SOX10	AAGCTCTGGAGGCTGCTG	CTTCCCGTTCTTCCGCCG	129 bp
GAPDH	TGGTATCGTGGAAGGACTCATG	AGTAGAGGCAGGGATGATGTTC	130 bp
ACTB	GAACGGTGAAGGTGACAGCAG	AGGATGGCAAGGGACTTCCTG	145 bp

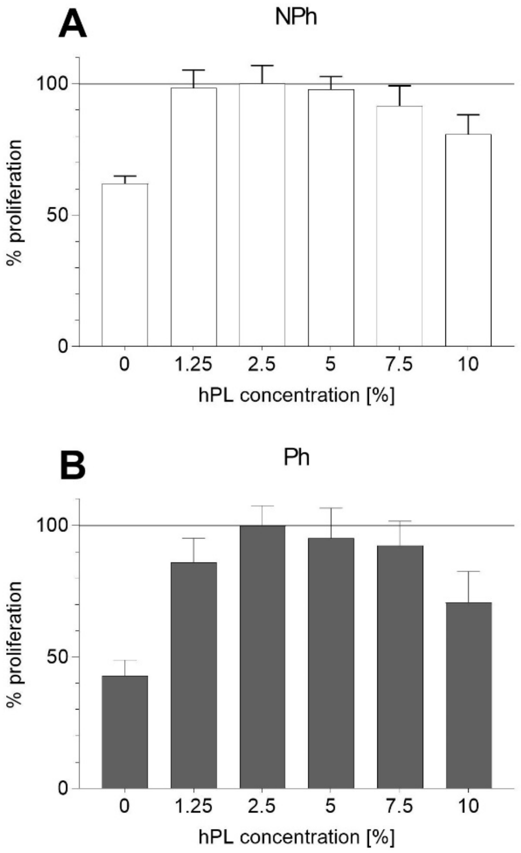

Figure S1: **percentage of proliferation for MUG Mel3 cell lines cultivated with different hPL concentrations.** A: MUG Mel3 NPh and B: MUG Mel3 Ph. OD-values highest at 2.5% hPL concentration were used as 100% proliferation. This experiment was conducted only once in six replicates and MTS was measured at 72 h.

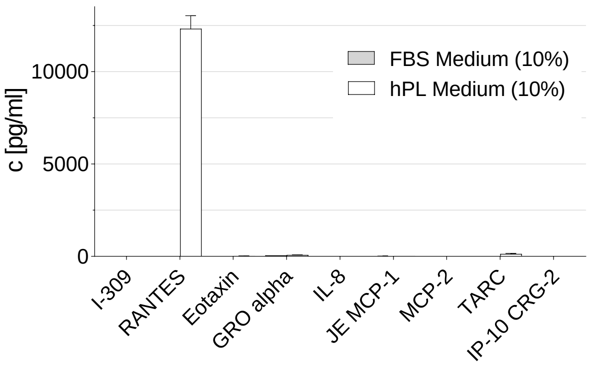

Figure S2: Human chemokine levels in media used for MUG Mel3 cell cultures. Comparison of FBS and hPL supplemented media.

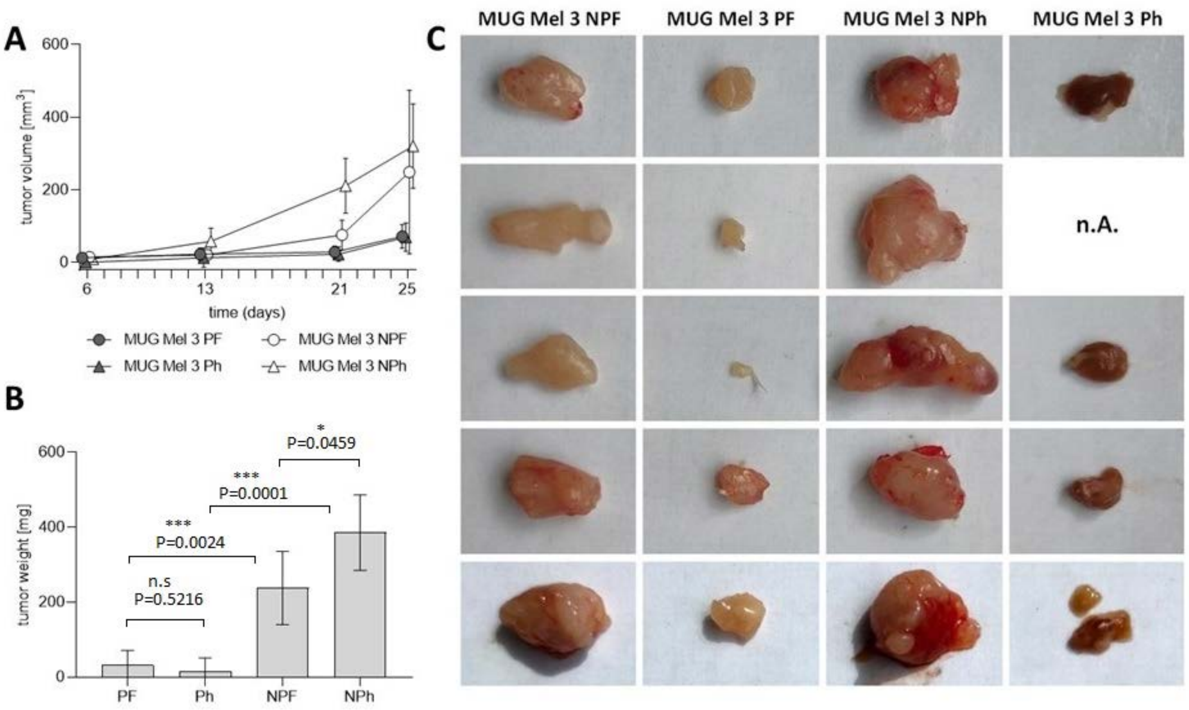

Figure S3: Tumorigenic profile of all four cell lines in immunodeficient NXG mice. Tumor volume [mm^3] over time, significance was tested by comparing culture conditions (FBS or hPL) within cell line origin (pigmented or non-pigmented) and the origins of cells within the same cultivation method on day of sacrifice: MUG Mel3 NPh/MUG Mel3 NPF p-value=0.6413; MUG Mel3 NPh/MUG Mel3 Ph p-value=0.0002; MUG Mel3 PF/MUG Mel3 Ph p-value=0.9296 and MUG Mel3 NPF/MUG Mel3 PF p value=0.0868. (A)tumor weight [mg] on day of sacrifice(B) are illustrated. Excised tumors of all mice are shown in C (n=5). High frequency ultrasound (HF-US) images of one representative mouse are presented in D. The images on day six were recorded with a 50 MHz (MX700) transducer; the images on day 25 with a 40 MHz (MX550D) transducer.

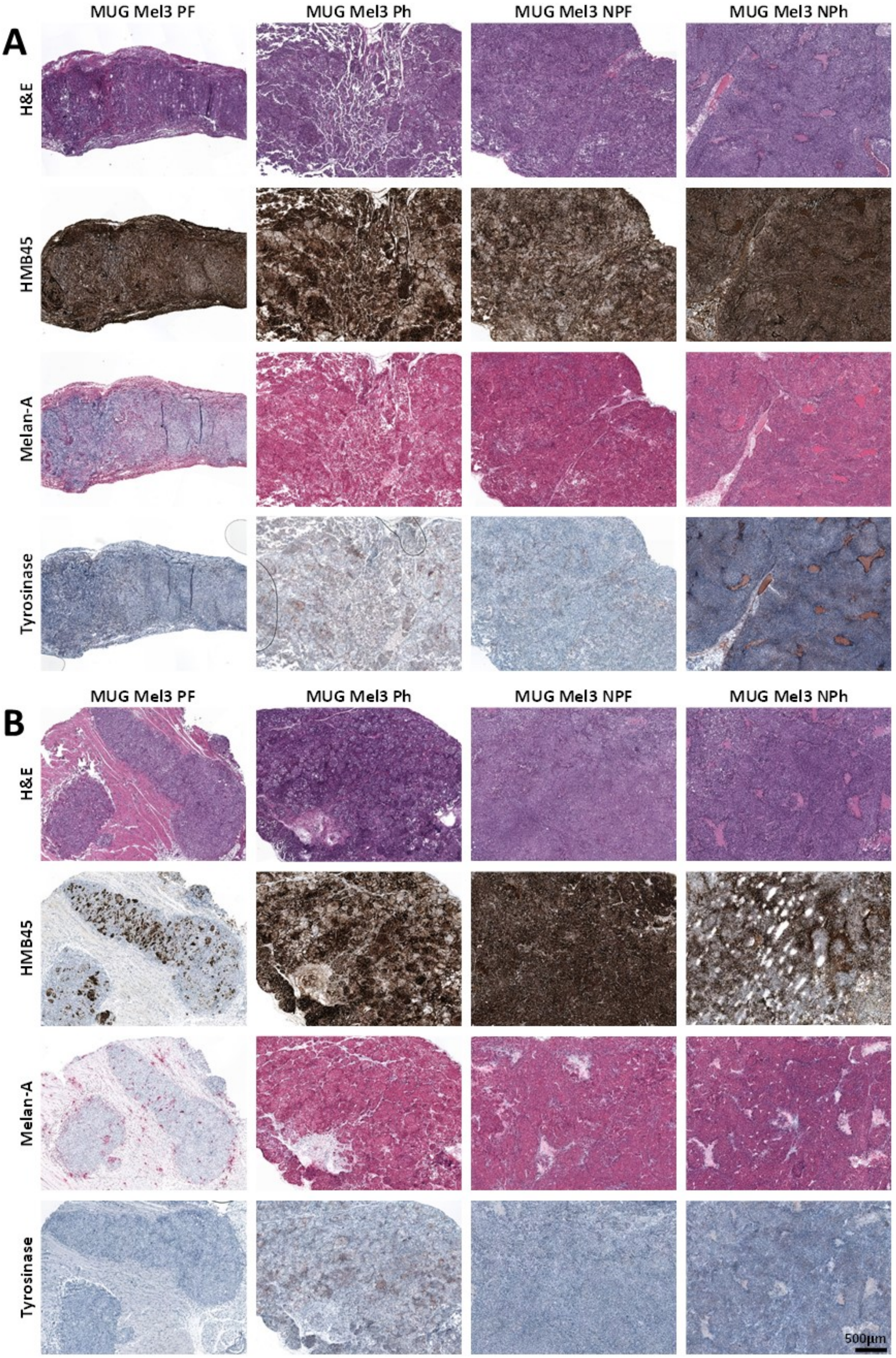
A
B
MUG Mel3 PF
MUG Mel3 Ph
MUG Mel3 NPF
MUG Mel3 NPh
H&E
HMB45
Melan-A
Tyrosinase
500µm

Figure S4: IHC of MUG Mel3 cell lines grown in CR ATH HO nude mice (A) and NXG mice (B). Tumor samples of one representative mouse are depicted showing staining for H&E, HMB45, Melan-A, and Tyrosinase.

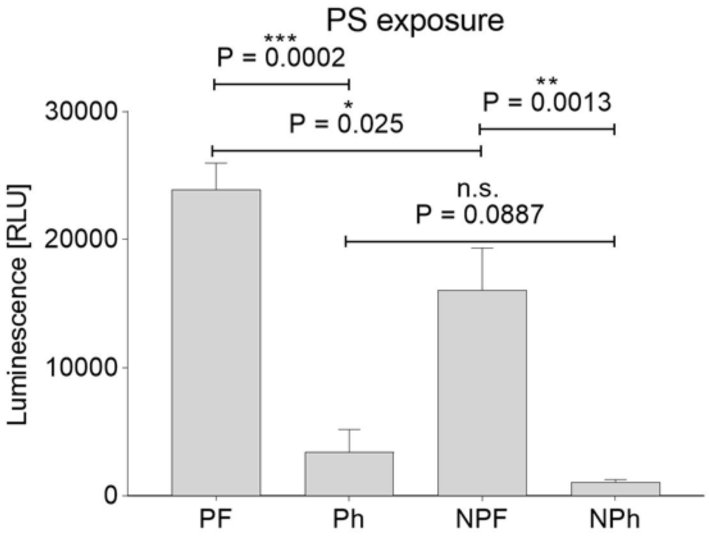

Figure S5: Expression of PS exposure measured by luminescence. PS exposure was measured using the RealTime-Glo™ Annexin V Apoptosis Assay from Promega. Measured PS levels of MUG Mel 3 were statistically significant lower in hPL containing medium compared to FBS. Experiments were performed at least three times. RLU: relative Luminescence.

.
